# Transcriptome and proteome profiling of adventitious root development in hybrid larch (*Larix kaempferi × Larix olgensis*)

**DOI:** 10.1186/s12870-014-0305-4

**Published:** 2014-11-26

**Authors:** Hua Han, Xiaomei Sun, Yunhui Xie, Jian Feng, Shougong Zhang

**Affiliations:** State Key Laboratory of Tree Genetics and Breeding, Chinese Academy of Forestry, Xiangshan Rd, Beijing, 100091 P. R. China; Research Institute of Forestry, Chinese Academy of Forestry, Xiangshan Rd, Beijing, 100091 P. R. China; Forestry Biotechnology and Analysis Test Center, Liaoning Academy of Forestry Sciences, Chongshan Rd, Liaoning, 110032 P. R. China

**Keywords:** Adventitious root development, Hybrid larch, 454 sequencing, 2D-DIGE, MALDI-TOF/TOF-MS, Polyamine synthesis, Stress response

## Abstract

**Background:**

Hybrids of larch (*Larix kaempferi × Larix olgensis*) are important afforestation species in northeastern China. They are routinely propagated via rooted stem cuttings. Despite the importance of rooting, little is known about the regulation of adventitious root development in larch hybrids. 454 GS FLX Titanium technology represents a new method for characterizing the transcriptomes of non-model species. This method can be used to identify differentially expressed genes, and then two-dimensional difference gel electrophoresis (2D-DIGE) and matrix-assisted laser desorption-ionization time-of-flight mass spectrometry (MALDI-TOF/TOF MS) analyses can be used to analyze their corresponding proteins. In this study, we analyzed semi-lignified cuttings of two clones of *L. kaempferi × L. olgensis* with different rooting capacities to study the molecular basis of adventitious root development.

**Results:**

We analyzed two clones; clone 25-5, with strong rooting capacity, and clone 23-12, with weak rooting capacity. We constructed four cDNA libraries from 25-5 and 23-12 at two development stages. Sequencing was conducted using the 454 pyrosequencing platform. A total of 957832 raw reads was produced; 95.07% were high-quality reads, and were assembled into 45137 contigs and 61647 singletons. The functions of the unigenes, as indicated by their Gene Ontology annotation, included diverse roles in the molecular functions, biological processes, and cellular component categories. We analyzed 75 protein spots (-fold change ≥2, *P* ≤0.05) by 2D-DIGE, and identified the differentially expressed proteins using MALDI-TOF/TOF MS. A joint analysis of transcriptome and proteome showed genes related to two pathways, polyamine synthesis and stress response, might play an important role on adventitious root development.

**Conclusions:**

These results provide fundamental and important information for research on the molecular mechanism of adventitious root development. We also demonstrated for the first time the combined use of two important technologies as a powerful approach to advance research on non-model, but otherwise important, larch species.

**Electronic supplementary material:**

The online version of this article (doi:10.1186/s12870-014-0305-4) contains supplementary material, which is available to authorized users.

## Background

Larches are very useful for afforestation because of their fast juvenile growth [1]. Among the various larches, hybrids of *Larix kaempferi × Larix olgensis* are widely planted in the mountainous regions of northeastern China for their high-quality timber, their pest resistance, and their amenity value [2]. The use of rooted stem cuttings is becoming the most popular method to propagate these hybrids. Adventitious root formation is a key step for stem cuttings, and it is a complex process affected by many factors [3-6]. Despite the ecological and economical importance of larch, little genomic research has been conducted on this species. It would be very useful to understand the biological process of adventitious root formation and development in larch.

In our previous study, two clones of *L. kaempferi × L. olgensis*, 25-5 and 23-12, are obviously different on the rooting rates, which were 100.0%, 94.7%, 93.3%, 96.3% and 25.1%, 19.6%, 21.5%, 22.9%, in 2001, 2002, 2003, and 2007, respectively. So, the two clones are good materials for studying the biological process of adventitious root formation. Additionally, we had examined the development process of adventitious roots in semi-lignified cuttings of hybrid larch [2], which consisted of three key stages: 14-18 days after cutting (DAC) for cell dedifferentiation and division; 25-35 DAC for meristem formation and development; 50-55 DAC for root formation and elongation. The early stages, 14 and 25 DAC, were crucial for the expression and regulation of rooting-related genes. These anatomical and physiological researches on adventitious roots development in hybrid larch make the further study on the molecular mechanisms governing adventitious root development feasible.

Although adventitious root formation is the key developmental process for asexual propagation of most plants, the molecular mechanisms of adventitious root formation are still poorly understood [7], because adventitious root formation is a complex quantitative genetic trait regulated by both environmental and endogenous factors. Only a limited number of molecular studies of adventitious root formation have been performed. Brinker et al. [8] identified 220 differentially expressed genes during different developmental stages of adventitious root formation in *Pinus contorta* hypocotyls which were treated with the auxin indole-3-butyric acid. Butler et al. [9] focused on gene expression patterns during adventitious root formation, and a number of mRNAs during adventitious root formation in apple were identified. Sorin et al. [10] identified some molecular markers which positively or negatively correlated with adventitious root formation by a proteomic analysis of different mutant genotypes of Arabidopsis. Chen et al. [11] provided a method suitable to study *de novo* root organogenesis. Ahkami et al. [12] identify specific genes determining the initiation and formation of adventitious roots by a microarray-based transcriptome analysis in the stem base of the cuttings of *Petunia hybrida* (line W115). Subramaniyam et al. [13] obtained a comprehensive transcript expression profiling for adventitious roots of Panax ginseng Meyer. However, very few genes were characterized for adventitious root formation of larch, due to the limited genome information.

RNA sequencing represents a powerful and rapid method for obtaining functional information on a genome-wide scale, especially for the large genomes and non-model species. Wu et al. [14] studied gene expression in North American ginseng root samples at seven developmental stages by 454 sequencing. Their results suggested that ginsenoside biosynthesis occurs at distinct stages of development. Firon et al. [15] identified the molecular mechanisms involved in the initiation of storage root formation in sweet potato by RNA sequencing. Alagna et al. [16] enriched the very small amount of sequence data currently available for olive, and identified genes involved in fruit quality via 454 pyrosequencing. Extensive analyses of transcriptome profiles [17-19] have identified functional genes with roles in specific biological processes. However, changes in mRNA transcript levels do not always reflect changes in protein levels, and there are many ways in which cellular events are regulated at the protein level. Therefore, a complementary protein analysis is necessary to clarify the molecular events at particular stages of development. Two-dimensional difference gel electrophoresis (2D-DIGE) combined with matrix-assisted laser desorption-ionization time-of-flight mass spectrometry (MALDI-TOF/TOF MS) has many advantages for protein analysis. Li et al. [20] detected significant changes in the abundance of certain proteins in cucumber roots in response to hypoxia using 2D-DIGE. Yan et al. [21] used a proteomic analysis to study the responses to growth restriction in maize plants supplied with sufficient nitrogen. Wang et al. [22] identified proteins involved in brassinosteroid regulation of rice growth using 2D-DIGE followed by MS. The results of those studies illustrated that transcriptomic or proteomic analyses are effective methods to find genes and proteins involved in regulating biological processes. Yang et al. [23] combined use of proteomic and transcriptomic analyses of M14 leaves and roots under NaCl treatment in sugar beet, these analyses revealed candidate genes and proteins for detailed functional characterization.

In this study, to gain a better understanding of adventitious root development in hybrid larch, we evaluated the transcriptomic profiles of two clones with different rooting capacity at two important stages of adventitious root development. Some of the genes identified as being differentially expressed between the two clones were further analyzed at the protein level. These data can remarkably expand the transcriptome and proteome database of larch, and provides a valuable platform to understand the molecular basis of regulation of adventitious root development in larch and other conifers.

## Results and discussion

### 454 sequencing

**Figure 1:**
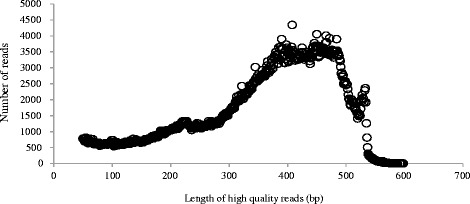
**Distribution of lengths of high-quality reads obtained by 454 sequencing.**

Compared with other techniques, the 454 pyrosequencing technique is a better alternative for *de novo* sequencing and analysis of the transcriptomes of non-model plants, because of the high number of reads generated per run and the low sequencing error rate in the obtained contigs [24]. This technology has been used to construct a number of expressed sequence tag (EST) libraries from plants including including maize [25], chestnut [24], olive [16], North American ginseng [14], and sweet potato [15]. Zhang et al. [26] used 454 sequencing to analyze Japanese larch during somatic embryogenesis. In this study, we constructed four cDNA libraries from two clones at two important stages of adventitious root development. Each of the cDNA libraries was sequenced using the 454 GS FLX Titanium platform. A total of 957,832 raw reads were produced from the four cDNA libraries. Most of the raw reads were between 450 and 500 bp long. After trimming adapter sequences and removing short sequences of less than 50 bases, a total of 910,607 high-quality reads were generated from the four cDNA libraries. Therefore, 95.07% of raw reads were useful. The length distribution of these high-quality reads is shown in Figure 1. Approximately 90% of the reads were between 300 and 500 bp in length.

**Figure 2:**
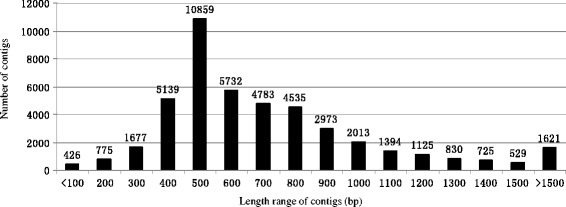
**Distribution of contigs length range.**

These 910,607 high-quality reads were assembled into 45137 contigs (a contig is a set of overlapping RNA segments derived from a single genetic source) and 61647 singletons (A single gene that identifies the comparable region in a target genome, but does not cluster with other genes to form a segment is called a singleton). The distribution of contig lengths is shown in Figure 2; 68.79% (31048) of contigs were 300–800 bp long, and 24.06% (10859) of contigs were 400–500 bp long. The median contig length was 739 bp, while the average contig length was 663 bp. The lengths of unigenes were similar to those obtained in previous transcriptome analyses of *Artemisia* [27], chestnut [24], American ginseng [28], and *Cucurbita pepo* [29]. The data shown here represent the first large-scale effort to generate cDNA resources and analyze the transcriptomes of hybrid larch during adventitious root development. These resources have been made publically available; the raw data have been submitted to the Short Read Archive (SRA) division of the Genbank repository (accession number SRP015266). The transcriptomic sequences should be useful for gene discovery in larch, and will be helpful for elucidating the genomic basis of adventitious root development.

### Functional annotation and classification

All unique sequences were first compared with sequences in the non-redundant database (nt) of the NCBI using the BLASTN algorithm. Then, they were compared with sequences in the two major public protein databases (Methods) using the BLASTX algorithm. When the E-value cutoff was set at 10-5, a total of 25965 contig sequences were annotated, which accounted for 57.5% of all contig sequences.

In principle, the higher the number of reads assembled in a contig, the higher the number of mRNA molecules encoding that gene in a given tissue sample. The read numbers of more than 700 are shown in Additional file 1. Among of the 58 contigs, 4 were involved in cell wall remodeling during plant growth and development, 10 were involved in the adaptive stress response and regulation of expression of many stress-responsive genes, and 2 were involved in polyamine synthesis. The others were involved in protein translation, synthesis, and degradation. Plant cell walls play many critical roles during plant growth, including regulation of cell differentiation, intercellular adhesion and communication, and defense against invasions by pests and pathogens [30-33], knowing which genes are involved in the formation and remodeling of plant cell walls is of great importance. Cell wall remodeling-related genes detected in this study should be related to the wound of cuttings. Stress-related genes could enhance survival of various cell types against stress. Polyamines are key regulators in cell growth and differentiation, the detection of polyamine synthesis-related genes should be related to the different development stages of adventitious root. The detailed discussion of stress-related genes and polyamine synthesis-related genes could be found in the following sections.

**Figure 3:**
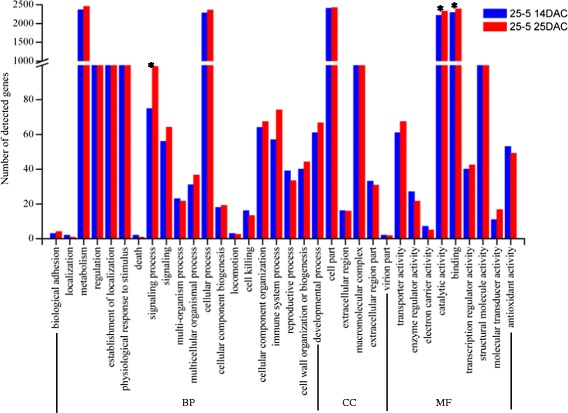
**Histogram showing Gene Ontology classifications of putative molecular function of unigenes from clone 25-5.** DAC, days after cutting. “*”, significant difference between 14 and 25 DAC, p value ≤0.05(calculated by chi-square test).

**Figure 4:**
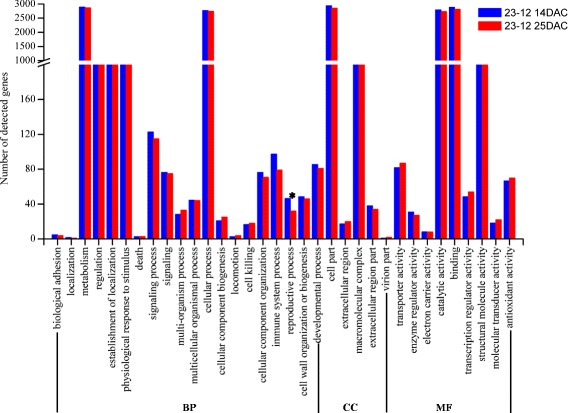
**Histogram showing Gene Ontology classifications of putative molecular function of unigenes from clone 23-12.** DAC, days after cutting. “*”, significant difference between 14 and 25 DAC, p value ≤0.05(calculated by chi-square test).

We used the Gene Ontology (GO) classification system to determine the possible functions of tagged genes. The GO ontology analysis showed that the distributions of gene functions for cDNA sequences from 25-5 and 23-12 on 14 days after cutting (DAC) and 25 DAC were similar (Figures 3 and 4). The functions of the identified genes covered various biological processes. Among 106784 unigenes (45137 contigs +61647 singletons), 13316 unigenes were classified into the biological process (BP) category, 5590 unigenes were classified into cellular component (CC), and 10885 unigenes were classified into molecular function (MF). The BP, CC, and MF categories in the GO classification system contained 34 subgroups (Figures 3 and 4), which covered the whole profile of gene expression in hybrid larch. The numbers of genes involved in some categories were significant difference between 14 DAC and 25 DAC (Figures 3 and 4). For 25-5 (Figures 3), the “catalytic activity” and “binding” genes were detected increased at 25 DAC than 14 DAC (p value were 0.0307 and 0.0484, Additional file 2), which was almost no change for 23-12 (Figures 4). “Catalytic activity” and “binding” genes, involved in many biological processes, were detected increased at adventitious root meristem formation stage (25DAC), which would play an important effect on root formation. For 23-12 (Figures 4), the “reproductive process” genes were detected significant decrease at 25 DAC (p value was 0.0335), which was almost no change for 25-5 (Figures 3). Although the “immune system process” genes have no significant changes between 14 DAC and 25 DAC for 25-5 and 23-12 separately, the genes increased 29.8% at 25 DAC for 25-5, decreased 18.6% at 25 DAC for 23-12, which would be better for 25-5 to form adventitious roots. Adventitious root formation is a complex process regulated by many factors, the “signaling process” genes were significant increase at 25 DAC for 25-5 (p value was 0.0241), but almost no change for 23-12, which indicated signaling process involved in regulation of adventitious root meristem formation (25DAC). The detailed information of the number of detected genes between 14 DAC and 25 DAC and the p values of each GO category were shown in Additional file 2.

### Differentially expressed proteins

**Figure 5:**
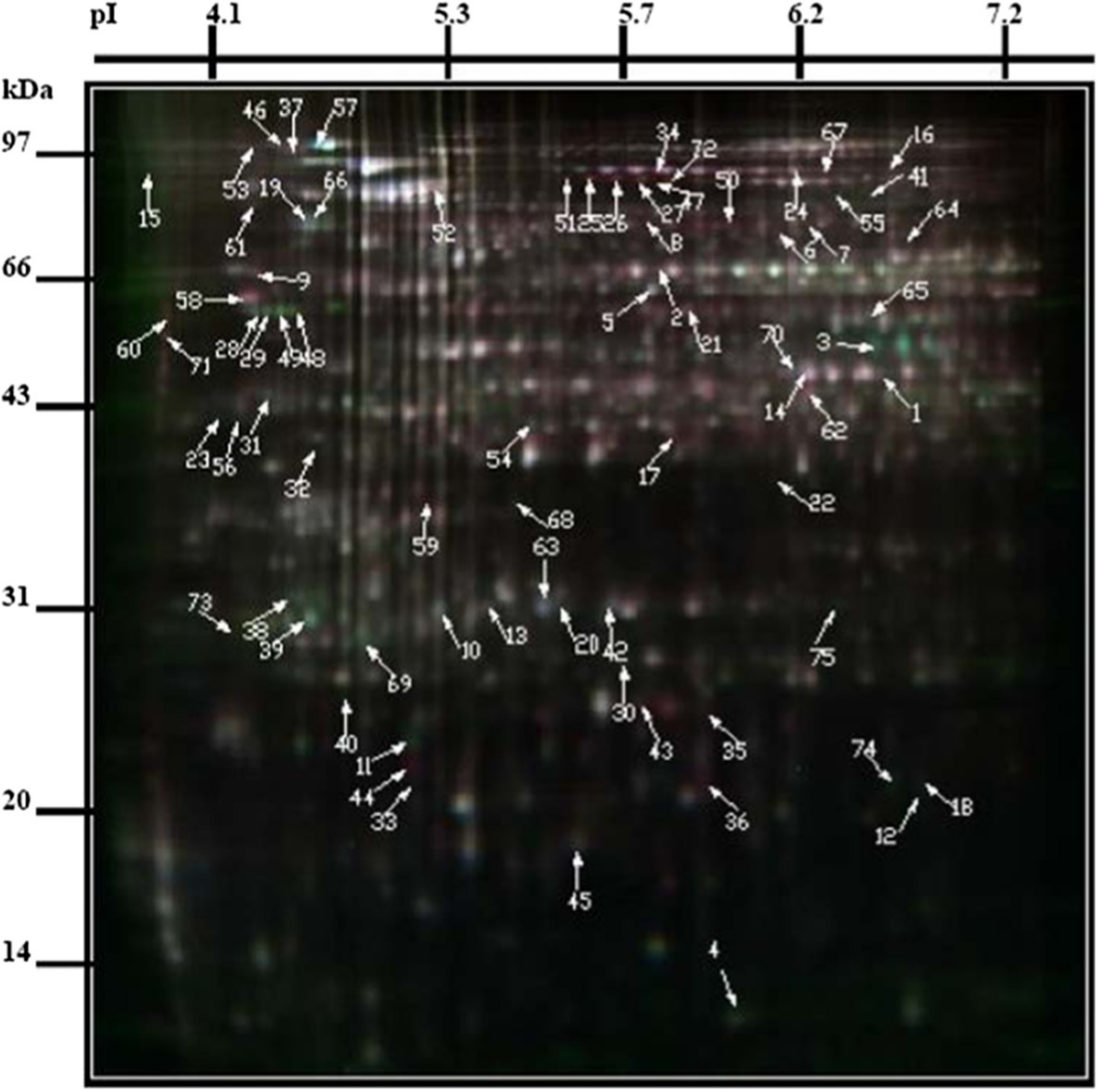
**Representative 2D-DIGE gel of 25-5 and 23-12 of** ***L. kaempferi × L. olgensis*** **.** Proteins were separated as described in Materials and Methods. Eighty-six differentially expressed proteins (fold change ≥ 2, *P* ≤ 0.05) were detected by 2D-DIGE and subsequently analyzed using DeCyder™ software. Seventy-five proteins identified successfully by matrix-assisted laser desorption-ionization time-of-flight mass spectrometry (MALDI-TOF/TOF-MS) are shown on this gel. Arrows indicate differentially expressed proteins between 25-5 and 23-12; numbers following arrows correspond to Additional file 3. Each spot on gel represents a protein. Molecular mass is shown on vertical axis; isoelectric point is shown in horizontal axis.

The profiles of proteins extracted from cutting tissue on 14 DAC, 25 DAC, and 35 DAC were investigated using a proteomics approach. We detected 86 differentially expressed proteins (fold changes ≥2, *P* ≤0.05) by 2D-DIGE. These proteins were subsequently analyzed using DeCyder™ software (Figure 5, spots 1–75). Approximately one-third of the differentially expressed proteins (32.4%) were present at 25 DAC (the initial cell formation stage in adventitious roots). We successfully identified 75 proteins using MALDI-TOF/TOF-MS. Detailed information for these proteins are shown in Additional file 3. For most of the proteins, the theoretical molecular mass (MM) and calculated isoelectric point (pI) was consistent with experimental data, as judged from the location of spots on 2D-DIGE gels (Figure 5; Additional file 3). However, there were some exceptions, such as spots 7, 8, 9, 15, 29, 41, and 66, which had theoretical MMs greater than the corresponding identified proteins, and spots 32, 37, 48, 49, 54, and 71, which had theoretical MMs smaller than the corresponding identified proteins (see Additional file 3). Several proteins, had the same functional annotation, showed in several different spots (spots 10, 11, 25, 26, and 27) on the gel (Figure 5). This phenomenon has also been reported in previous studies [34]. There are several possible explanations for this, including post-translational modification, protein degradation, different encoding genes, and protein translation from alternatively spliced mRNAs. Another possibility is that these spots represented protein homologs unique to larch.

**Figure 6:**
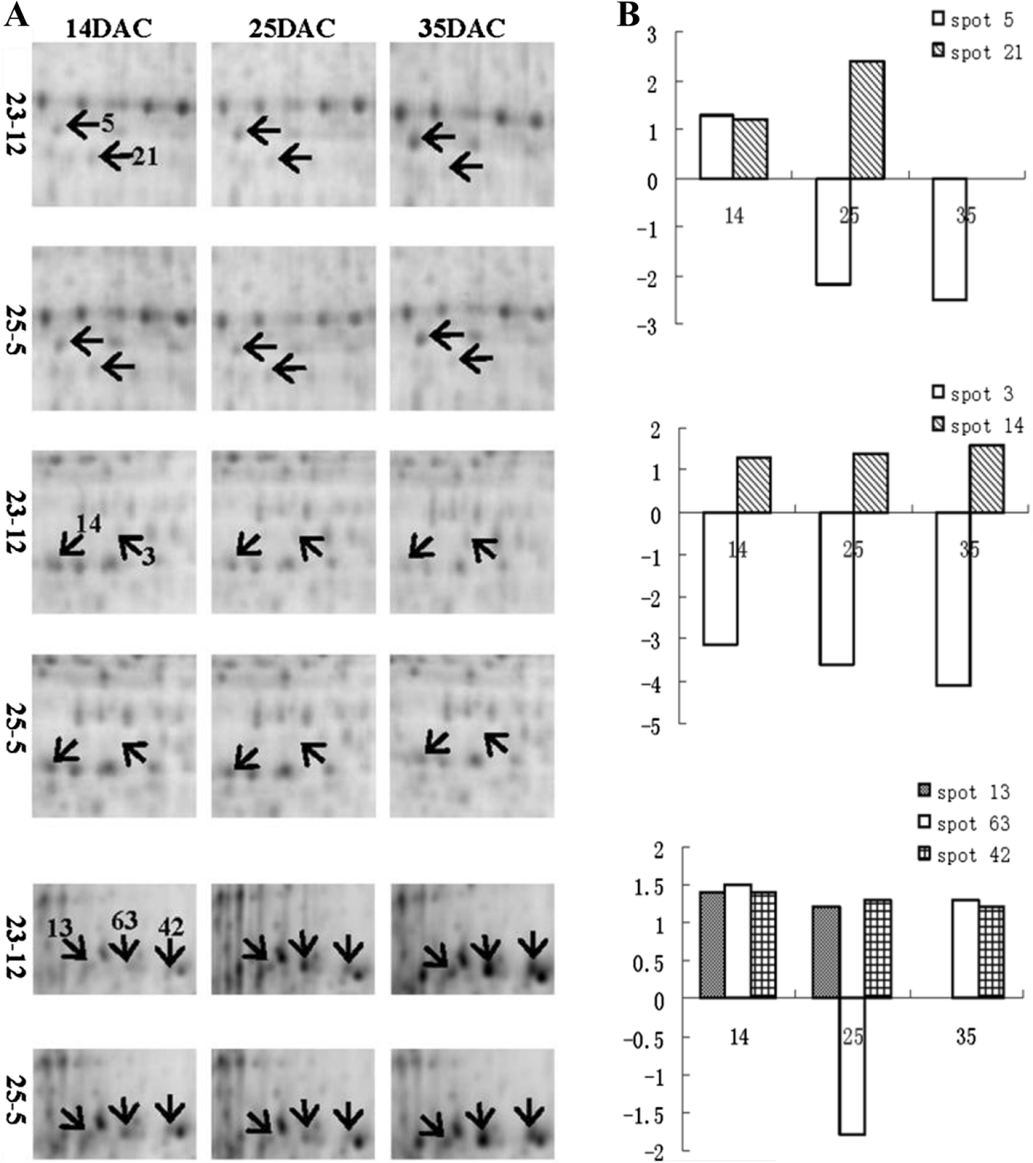
**Differentially expressed proteins at various stages of adventitious root development. (A)** Protein spots on the gels. Some of the proteins shown in Figure 5 that were differentially expressed between 23-12 and 25-5 at 14 DAC, 25 DAC, and 35 DAC. **(B)** Fold changes of the differentially expressed proteins. Bar plot showing -fold changes in protein abundance between 23-12 and 25-5. Vertical axis shows -fold change; horizontal axis shows DAC. Value for -fold change was calculated by comparing abundance in 25-5 with that in 23-12, “-” indicates down-regulation. DAC: days after cutting.

The 75 identified proteins could be divided into eight categories according to their functional annotation (see Additional file 3). Of these proteins, 14.7% were involved in carbohydrate and energy metabolism, 6.7% in protein translation and degradation, 34.7% in cell defense, 9.3% in hormone-related activities, 5.3% in signal transduction, 5.3% in RNA transcription and processing, and a few were involved in other metabolic pathways. Proteins of unknown function made up 24% of the 75 identified proteins. Figure 6A,B shows the abundance of some of the differentially expressed proteins at different stages of adventitious root development.

One notable result was that the expression patterns of S-adenosylmethionine synthetase 2 (SAMS2, spot 21, Figure 6A,B) and S-adenosyl methionine decarboxylase (SAMDC, spot 22), which are involved in polyamine synthesis, and a late-embryogenesis-abundant protein (spot 33) and an intracellular pathogenesis-related protein (spot 45), which are involved in stress responses, were the same as those detected for their encoding genes in the transcriptomic analysis. The detailed discussions of the effects of these genes on adventitious root development are showed in “elucidation of important signaling pathways” part.

### Elucidation of important signaling pathways

#### Polyamine synthesis pathway

Polyamines are small polycationic molecules; the main polyamines in living organisms are putrescine (Put), spermidine (Spd), and spermine (Spm) [35]. They are found ubiquitously in living organisms and function in a wide variety of biological processes. Polyamine biosynthesis involves a series of steps catalyzed by specific enzymes [36,37], including SAMDC and SAMS. In this study, the contigs UN_lyscdhit99_32363 and UN_lyscdhit99_25323 were annotated as SAMS2 and SAMDC proenzymes, respectively, in the transcriptome data (Table 1). The read numbers assembled in these two genes in 25-5 (strong rooting capacity) were higher than those in 23-12 (weak rooting capacity) at the root cell dedifferentiation and division stage (14 DAC) and at the root initial cell formation stage (25 DAC). In the proteomic profile, two proteins were identified as SAMS2 (spot 21) and SAMDC (spot 22); importantly, these two proteins were expressed at higher levels in 25-5 than in 23-12 on both 14 DAC and 25 DAC (Table 1). The SAMDC protein was also up-regulated at 35 DAC (see Additional file 3). This result that polyamines play a role in regulating root development is consistent with the results of other studies. For example, Tisi et al. [38] showed that polyamine catabolism strongly affected root development and xylem differentiation in maize (*Zea mays*). Niemi et al. [39] showed that polyamines accelerated adventitious root formation and increased subsequent root growth of Scots pine (*Pinus sylvestris* L.) hypocotyl cuttings *in vitro*. Tang and Newton [40] demonstrated that polyamines promoted root elongation and growth in regenerated Virginia pine plantlets. In other studies, polyamines were shown to improve the rooting rate [41], regulate vascular development [42], and enhance tolerance of the root to salinity-alkalinity stress [43]. In this study, the expression of SAMDC and SAMS2 at the transcriptional and translational levels demonstrated that polyamines play an important role in regulating adventitious root development in hybrid larch, possibly via increasing root cell division and enhancing tolerance of the roots to stresses.

**Table 1:** **Genes and proteins related to polyamine synthesis and the stress response**

		**25-5/23-12**		
**Gene**		**14 DAC**	**25 DAC**	
Query name	NR annotation	Relative read number		Species
UN_lyscdhit99_32363	S-adenosylmethionine synthase 2	322/180	380/303	*Pinus contorta*
UN_lyscdhit99_25323	S-adenosylmethionine decarboxylase proenzyme	205/150	346/227	*Vitis vinifera*
UN_lyscdhit99_17285	Embryo-abundant protein	39173/18594	33033/37185	*Picea abies*
UN_lyscdhit99_23395	Putative intracellular pathogenesis-related protein	518/206	147/343	*Picea glauca*
Protein				
Spot no.	Protein name	-fold change		
21	S-adenosylmethionine synthetase 2	1.2	2.4	*Pinus contorta*
22	S-adenosylmethionine decarboxylase	1.3	2.4	*Pisum sativum*
33	Late embryogenesis abundant protein	2.8	1.2	*Picea glauca*
45	Intracellular pathogenesis-related protein	3.5		*Picea glauca*

*Abbreviations: DAC* days after cutting.

25-5, clone with strong rooting capacity; 23-12, clone with weak rooting capacity.

“Read number”, number of reads assembled in a contig; the read number had been normalized to the total read number (Additional file 5).

“-fold change” compares expression level in 25-5 with that in 23-12.

Number in “spot no.” corresponds to Figure 5 and Additional file 3.

“Species” the annotation information come from which plants.

#### Stress response pathway

Cuttings excised from plants are subjected to several stresses, such as wounding and pathogen attack. The responses of cuttings to stresses are complex and involve a number of metabolic changes to protect cells and macromolecules. In this study, Contigs of UN_lyscdhit99_17285 and UN_lyscdhit99_23395 were annotated as an embryo-abundant protein and a putative intracellular pathogenesis-related protein, respectively (Table 1). Both of these proteins are related to various stress responses. We identified corresponding proteins in the proteomic profile; the embryo-abundant protein corresponded to spot 33, and the intracellular pathogenesis-related protein corresponded to spot 45.

The late embryogenesis abundant (LEA) protein family is a large family that includes proteins that accumulate at late stages of seed development or in vegetative tissues in response to some stresses. Many studies have shown that LEA proteins have important effects on salt tolerance in plants. Aghaei et al. [44] showed that a LEA protein was up-regulated in the hypocotyl and root of soybean treated with 100 mM NaCl. Zhang et al. [45] identified a putative salt-tolerance gene *LEA1* in *Thellungiella salsuginea*, and confirmed that TsLEA1 conferred salt tolerance when expressed in yeast and transgenic *Arabidopsis*. Bai et al. [46] demonstrated that transgenic tobacco expressing the LEA3-1 protein encoded by a gene from *Medicago sativa* showed enhanced salt tolerance. Park et al. [47] showed that LEA14 might positively regulate the response to dehydration by enhancing root cell lignification in sweet potato. Overexpression of SmLEA showed faster root elongation and a higher salt and drought tolerance in *Salvia miltiorrhiza* [48]. In this study, a LEA protein was annotated in the transcriptome data. The read number assembled in this gene was higher in clone 25-5 (strong rooting capacity) than in clone 23-12 (weak rooting capacity) at the root cell dedifferentiation and division stage (14 DAC). Consistent with this, the corresponding protein was expressed at higher levels in 25-5 than in 23-12 at 14 DAC (Table 1). However, the read number assembled in *LEA* was lower in 25-5 than in 23-12 at the root initial cell formation stage (25 DAC), and there was no significant difference in the read number assembled in *LEA* between 25-5 and 23-12 at 35 DAC (see Additional file 3). Together, these findings conjectured that this LEA might positively regulate the response to various stresses by enhancing lignification of cuttings, mainly at the early stage of adventitious root development.

Pathogenesis-related (PR) proteins are plant proteins induced by abiotic and biotic stresses [49]. They accumulate around damaged cell walls to protect plants against infection by fungi, bacteria, or viruses [50]. Many studies have shown that PR proteins have important effects on root development. Bantignies et al. [51] showed that a PR-10-like protein was constitutively expressed at all stages of root development in roots of white lupin. Borghi et al. [52] showed that *PR* genes affected potassium homeostasis in *Arabidopsis thaliana*. Takeuchi et al. [53] identified a root-specific PR protein induced by drought and salt treatments in rice. Immunohistochemical analyses showed that this protein strongly accumulated in cortex cells around the vascular system of roots. Koehler et al. [54] identified PR proteins associated with cold tolerance in strawberry. The abundance of PRprotein1.2 significantly increased in wheat seminal roots after 7 days of waterlogging [55]. In this study, the expression of a PR protein at transcriptional and translational levels implied that it has a role in adventitious root development. It was significantly up-regulated in 25-5 (strong rooting capacity) at the adventitious root cell dedifferentiation and division stage (14 DAC) (Table 1). Therefore, it may play a crucial role in protecting the cuttings from damage caused by bacteria and fungi, mainly at the early stage after cutting, thus allowing normal adventitious root development.

## Conclusions

In the present study, two clones of hybrid larch, different on rooting capacity, were analyzed from RNA and protein level. A transcriptome database of adventitious root development was generated included a total of 957832 raw reads, which would be useful to future molecular studies of larch. The protein profile of adventitious root development was also analyzed, 75 proteins were identified, which enriched the protein database. A joint analysis of transcriptome and proteome showed.that genes related to polyamine synthesis and the stress response might play an important role on adventitious root development. This study provides fundamental and important information for subsequent studies on the molecular mechanism of adventitious roots development. Furthermore, we demonstrated for the first time the combined use of two important technologies as a powerful approach for studying non-model, but otherwise important, larch species.

## Methods

### Plant materials

**Figure 7:**
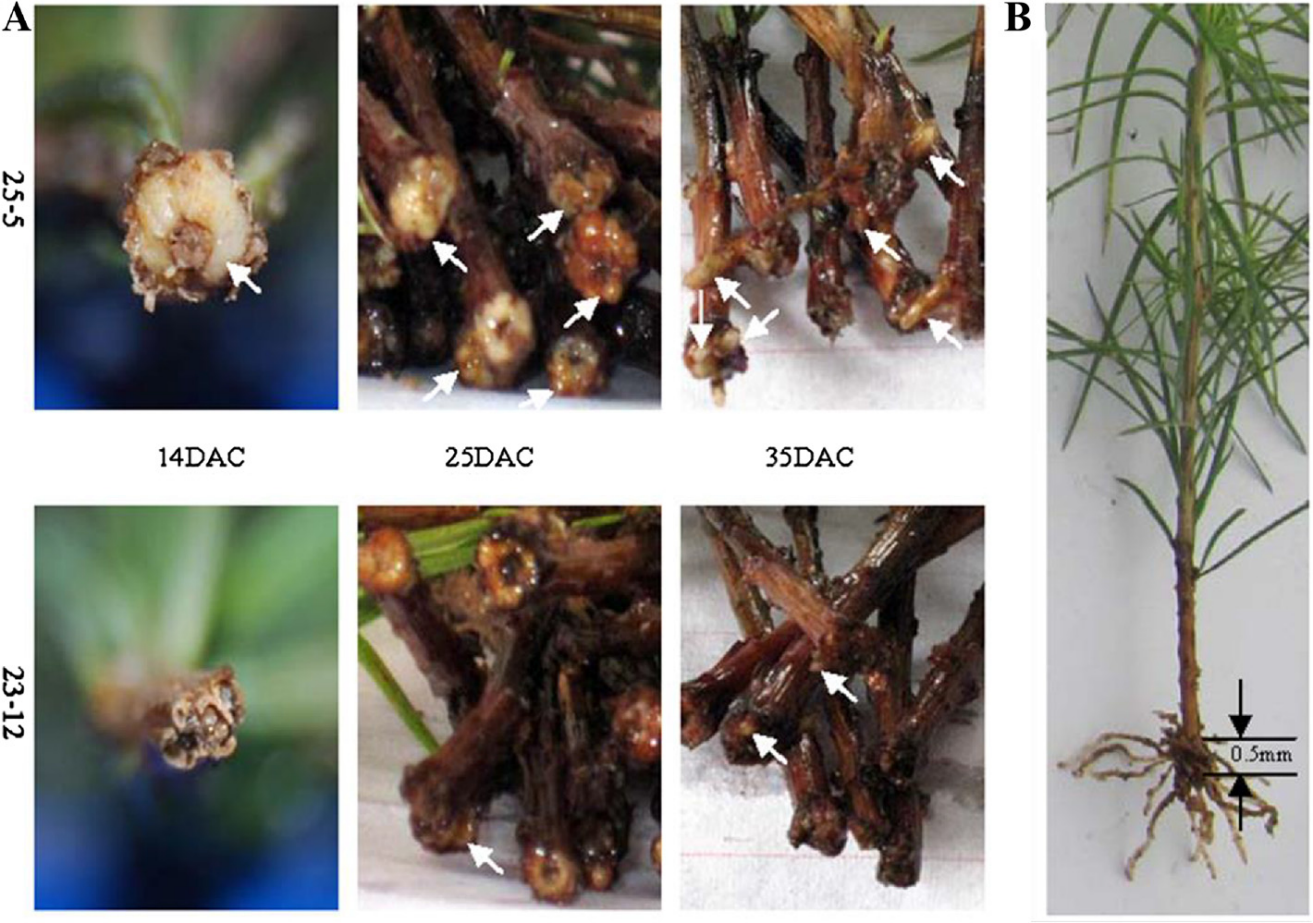
**Plant materials. (A)** Appearance of stem cuttings in clones 25-5 and 23-12. On 14 DAC, adventitious root cells began to dedifferentiate and divide, white callus (arrow) formed in the region between the xylem and epidermis at the base of cuttings in clone 25-5. Then, on 25 DAC, white protrusions (arrow), which later developed into adventitious roots, were located around callus after adventitious root initial cell formation. More white protrusions formed in clone 25-5 than in 23-12. By 35 DAC, young roots (arrow) protruded through the epidermis and began to elongate with the emergence and development of adventitious root primordia. More young roots formed in clone 25-5 than in clone 23-12. **(B)** Part of stem cutting used in this study. Adventitious roots formed at the bottom section (5-mm thick; arrow) of cuttings. Therefore, proteins and RNA were extracted from this part for analysis in this study. DAC: days after cutting.

In this study, two clones of *L. kaempferi* × *L. olgensis*, 25-5 (strong rooting capacity) and 23-12 (weak rooting capacity), and two early stages of adventitious root development, the root cell dedifferentiation and division stage (14 DAC) and the root initial cell formation stage (25 DAC) [2], are selected to study the molecular basis of developmental regulation of adventitious root formation. All plant material used in this study was propagated at Dagujia forest farm in Liaoning province in northeastern China (longitude of 42°2'48''N, latitude of 124°47'48''E; altitude of 225–394 m; and annual average temperature of 5.6°C) [2]. A completely randomized block design with three replicates was used. Four to five cuttings were randomly taken from each replicate, and cuttings from each replicate were separated according to clones and development stages. A 5-mm-thick portion from the bottom of each cutting was taken for RNA and protein extraction. Images of the stem cuttings at different stages of adventitious root development are shown in Figure 7A. The 5-mm-thick portion used for analyses is illustrated in Figure 7B.

Our experimental research complied with institutional, national, or international guidelines. Field studies were conducted in accordance with local legislation, and with the permissions of forestry administrative department. We did not use any endangered species and complied with the Convention on the Trade in Endangered Species of Wild Fauna and Flora. Voucher specimens (LH 2013001, LH 2013002, LH 2013003, LH 2013004), were identified by Prof. Xiaoshan Wang of the research institute of forestry, Bejing, and have been deposited in the herbarium of Chinese Academy of Forestry.

### RNA extraction and cDNA library construction

Total RNA was prepared by the method of Sánchez et al. [56]. The concentration and quality of total RNA were determined using a NanoDrop 1000 spectrophotometer (Thermo Scientific, Wilmington, DE, USA) and checked on 1% agarose gels before proceeding (see Additional file 4). Approximately 1 μg total RNA was converted into cDNA using a SMART cDNA synthesis kit (Clontech, Palo Alto, CA, USA), optimizing the conditions to obtain a large quantity of clean cDNA in a small volume. For second-strand synthesis, PCR was carried out using a small aliquot (1/10th volume) of the primary template and Advantage 2 Polymerase Mix (Clontech). The thermal cycling program was as follows: initial denaturation at 95°C for 60 s, followed by 18 cycles of denaturation at 95°C for 15 s and annealing at 65°C for 30 s, followed by extension at 68°C for 3 min. For each sample and all PCR reactions, the PCR products were purified using a PureLink PCRTM purification kit (Invitrogen, Carlsbad, CA, USA). Double-stranded cDNA was quantified with a spectrophotometer (NanoDrop 1000, Thermo Scientific). The products were checked on a 2% agarose gel to verify cDNA quality and fragment length.

### 454 sequencing and assembly

Digested cDNA was recovered with a QIAquick PCR Purification kit (Qiagen, Hilden, Germany). Approximately 7 μg ds cDNA was sheared via nebulization into small fragments, and sequenced using the GS FLX Titanium kit. Raw unprocessed EST sequences generated from this study have been submitted to the Short Read Archive (SRA) division of Genbank. The 454 SFF file containing raw sequences and sequence quality information can be accessed through the SRA web site under the accession number SRP015266. Using the GS FLX pyrosequencing software, we selected high-quality sequences (>99.5% accuracy on single base reads) for further processing and assembly. Adapter trimming and poly (A/T) and short sequence (<50 bp) removal were performed by in-house Perl scripts to obtain clean ESTs. We used Newbler software (provided with the Roche GS FLX sequencer) for sequence assembly. The quality score threshold was set at 40.

### Functional annotation and classification

The assembled unique transcripts were compared with the sequences in the non-redundant database of GenBank using the BLASTN algorithm to find and remove ribosomal RNA sequences [57]. The remaining sequences that putatively encoded proteins were searched against the NCBI non-redundant protein (Nr) database (http://www.ncbi.nlm.nih.gov/) using the BLASTX algorithm, and against the UniProt protein database (http://www.uniprot.org/help/uniprotkb) and the KEGG protein database (http://www.genome.jp/kegg/). A typical cut-off value of E <1.0-5 was used. The GO [58] system was used for functional classification of sequences. GO provides a structured and controlled vocabulary to describe gene products according to three categories: molecular function, biological process, and cellular component (http://geneontology.org/).

### Gene expression analysis

Comparison of gene expression between the strong rooting capacity clone and weak rooting capacity clone was done following the method of Audic et al. [59] and Barakat et al. [24].

### Normalization between the libraries

We did normalization between the libraries, including the comparison of the number of unigenes detected in each library of GO classification, and the total number of reads in each library when found the difference between two libraries. The detailed information of normalization was shown in Additional file 5.

### Protein extraction and preparation

For each sample, 2 g tissue was ground into a fine powder in liquid nitrogen for protein extraction. The lyophilized powder was homogenized in lysis buffer (7 M urea, 2 M thiourea, 4% 3-[(3-cholamidopropyl) dimethylammonio]-1-propanesulfonate (CHAPS), 10 mM Tris) for 30 min at room temperature with repeated shaking. The undissolved powder was removed from the homogenate by centrifugation at 40 000 × g for 60 min at 4°C. The supernatant was stored in aliquots at –80°C. Protein concentration was determined with a Bradford assay kit (BioRad, Hercules, CA, USA) using albumin diluted in lysis buffer as the internal standard.

### Protein labeling with CyDye DIGE fluor

Sample lysates were labeled with Cy2, Cy3, and Cy5 following the protocols described in the Ettan DIGE User Manual (18-1164-40 Edition AA, GE Healthcare, Buckinghamshire, UK). Typically, 50 μg lysate was labeled with 400 pmol Cy3 or Cy5, while the same volume of a pooled standard containing equal quantities of all samples was labeled with Cy2. Labeling reactions were carried out in the dark on ice for 30 min before quenching with 1 mL 10 mM lysine for 10 min on ice. These labeled samples were then combined for 2D-DIGE analysis.

### 2D-DIGE and image analysis

We conducted 2D-DIGE as follows: The IPG strips (24 cm, pH 3–10, and NL) were rehydrated with labeled samples in the dark overnight with rehydration buffer (7 M urea, 4% w/v CHAPS, 20 mM dithiothreitol (DTT), and 1% v/v IPG buffer with a trace amount of bromophenol blue). First-dimension IEF was performed using an Ettan IPGphor System (GE Healthcare) for a total of 67 kV h at 20°C. The strips were then treated with a two-step reduction and an alkylation step prior to the second-dimension SDS-PAGE. After equilibration with a solution containing 6 M urea, 30% glycerol, 2% sodium dodecyl sulfate (SDS), 50 mM Tris-Cl, pH 8.8, and 0.5% w/v DTT, the strips were treated with the same solution except that it contained 4.5% w/v iodoacetamide instead of DTT. The strips were overlaid onto 12% polyacrylamide gels (20 × 24 cm), immobilized to a low-fluorescence glass plate, and electrophoresed for 12–18 h at 30 mA per gel using an Ettan DALT Twelve System (GE Healthcare). The Cy2, Cy3, and Cy5-labeled images were acquired using a Typhoon 9410 scanner (GE Healthcare) at excitation/emission wavelengths of 488/520 nm, 532/580 nm, and 633/670 nm, respectively. The DIGE images were analyzed by DeCyder 6.5 following the Ettan DIGE User Manual (GE Healthcare). Spot detection was performed using the DIA (differential in-gel analysis) module with the estimated number of spots set to 2500. Only spots present in all three replicate gels and qualitatively consistent in size and shape were considered. After removing artifact spots by manual editing, the DIGE images were further analyzed using the DeCyder BVA (biological variation analysis) module. A 0.05 significance level was used to define differences between groups when analyzing parallel spots. One-way ANOVA and Student-Newman-Keuls tests were conducted using the SAS software package version 8.2 (SAS Institute).

### In-gel tryptic digestion and MALDI-TOF/TOF-MS analysis

Separate preparative gels were run to obtain sufficient amounts of protein for MS analysis. These gels were fixed and stained with colloidal Coomassie brilliant blue (CBB). Proteins of interest, as defined by the 2D- DIGE/DeCyder analysis, were excised from the CBB-stained gels for a modified in-gel tryptic digestion procedure. Gel pieces were first discolored in 50% acetonitrile and 25 mM ammonium bicarbonate, and then subjected to reduction and alkylation in 10 mM DTT and 55 mM iodoacetic acid, respectively. Following vacuum drying, the gel pieces were incubated with sequencing-grade modified trypsin (Promega, Madison, WI, USA) at a final concentration of 0.01 mg/mL in 25 mM ammonium bicarbonate for 16 h at 37°C. Supernatants were collected, vacuum dried, and then redissolved in 50% acetonitrile and 0.1% trifluoroacetic acid (TFA) for MS analysis using an ABI 4800 Proteomics Analyzer MALDI-TOF/TOF-MS. The TOF spectra were recorded in the positive ion reflector mode with a mass range from 700 to 4000 Da, and 10 of the strongest peaks in each sample were chosen for MS/MS analysis. The spectra were corrected by an external standard method using trypsin-treated myoglobin peptides. The MS/MS results were searched using GPS (Applied Biosystems, Foster City, CA, USA) - MASCOT (Matrix Science, London, UK) with the following criteria: NCBInr database; species restriction, green plants; MS tolerance was set at 6100 ppm and MS/MS at 60.6 Da; at most one missed cleavage site; fixed modification was carbamidomethyl (Cys) and variable modification was oxidation (Met); and cleavage by trypsin was on the C-terminal side of Lys and Arg unless the next residue was Pro. If peptides matched to multiple members of a protein family, or if a protein appeared under different names and accession numbers, the entry with the highest score was selected. In addition, the theoretical molecular weights and pI of the identified proteins were calculated using the Peptide Mass program (http://web.expasy.org/peptide_mass/).

### Availability of supporting data

The raw sequences data supporting the results of this article are available in the Short Read Archive (SRA) (accession number SRP015266), http://www.ncbi.nlm.nih.gov/sra.

## Additional files

Additional file 1:
**Detailed information for contigs with more than 700 reads.**
Additional file 2:
**The detailed information of the number of detected genes between 14 DAC and 25 DAC in clones 25-5 and 23-12, and the p values of each GO category.**
Additional file 3:
**Identification of differentially expressed proteins between clones 23-12 and 25-5 of** ***L. kaempferi*** **×** ***L. olgensis*** **using MALDI-TOF/TOF-MS.**
Additional file 4:
**Concentration and quality of total RNA extracted from materials used in this study.**
Additional file 5:
**The detailed information of normalization.**
