# Supplementary material for: Transcriptome and proteome profiling of adventitious root development in hybrid larch (Larix kaempferi × Larix olgensis)
Source: BMC Plant Biol. 2014 Nov 26;14:305. doi: 10.1186/s12870-014-0305-4 (PMC4253636; doi:10.1186/s12870-014-0305-4)
Supplement: Additional file 3: — Identification of differentially expressed proteins between clones 23-12 and 25-5 of L. kaempferi × L. olgensis using MALDI-TOF/TOF-MS. [file 12870_2014_305_MOESM3_ESM.pdf]

Table 1 Identification of differentially-expressed proteins between stem cuttings of the 23-12 and 25-5 clones of *L. kaempferi*×*L. olgensis* using MALDI-TOF/TOF-MS

Spot no.	Acession no.	Protein name	Theoretical MM(Kda)/pI	Experimental MM(Kda)/pI	NP	C (%)	Species	Fold change		
								14DAC	25DAC	35DAC
**Carbohydrate and Energy Metabolism**										
1	gi|56562181	Formate dehydrogenase	42.1/6.87	42-45/6.2-6.5	12	20	*Solanum lycopersicum*		2.1	
2	gi|1353640	Iisocitrate lyase	64.2/7.26	63-66/5.9-6.2	9	17	*Pinus taeda*	1.9	1.8	2.0
3	gi|22326811	Isocitrate dehydrogenase	54.1/8.05	50-55/6.6-7.1	10	17	*Arabidopsis thaliana*	-3.1	-3.6	-4.1
4	gi|20218815	Putative ribulose bisphosphate carboxylase small chain	17.7/7	10-14/6.0-6.3	4	18	*Pinus pinaster*	-1.8		
5	gi|4090843	Ribulose-1,5-bisphosphate carboxylase/oxygenase large subunit	51.7/6.2	55-64/5.8-6.1	10	23	*Cathaya argyrophylla*	1.3	-2.2	-2.5
6	gi|3790100	Pyrophosphate-dependent phosphofructokinase beta subunit	61.7/6.11	67-76/5.9-6.2	5	6	*Citrus x paradisi*	1.4	-1.4	
7	gi|3023685	2-phosphoglycerate dehydratase	47.6/5.41	66-73/5.9-6.1	11	33	*Alnus glutinosa*	1.7		
8	gi|3328122	Phosphoglycerate kinase precursor	50.4/7.68	66-73/5.8-6.0	10	24	*Solanumtuberosum*	2.1		
9	gi|3023813	Glyceraldehyde-3-phosphate dehydrogenase	36.6/6.41	60-66/4.6-4.9	10	21	*Taxus baccata*	1.4	2.4	1.7
10	gi|228017690	ATP synthase CF0 B subunit	20.9/5.18	24-28/5.1-5.3	7	38	*Pinus pinaster*		1.3	
11	gi|228017177	ATP synthase CF0 B subunit	20.9/5.54	20-24/5.2-5.5	7	26	*Pinus leiophylla*	2.3	2.3	2.7
**Protein Translation and Degradation**										
12	gi|624938	Ribosomal protein L16	20.8/9.97	20-22/7.0-7.4	10	48	*Arabidopsis thaliana*			
13	gi|162458463	Putative beta 4 proteasome subunit	23.1/5.4	25-29/5.2-5.4	7	32	*Zea mays*	1.4	1.2	
14	gi|57900269	F-box domain containing protein-like	39.3/6.86	38-45/6.1-6.3	10	33	*Oryza sativa*	1.3	1.4	1.6
15	gi|48210029	Kelch repeat-containing F-box family protein	56.8/4.93	79-85/3.5-4.1	12	26	*Solanum demissum*			
16	gi|3915866	Glutaminyl-tRNA synthetase	90.4/6.31	90-95/6.2-6.4	15	17	*Lupinus luteus*			
**Signal Transduction**										
17	gi|15239935	Phox (PX) domain-containing protein	46.5/6.76	42-47/5.9-6.1	14	41	*Arabidopsis thaliana*	1.7	2.4	2.0
18	gi|162464413	Rop3 small GTP binding protein	24.2/9.45	21-25/6.8-7.2	10	28	*Zea mays*	-1.7	-2.4	
19	gi|55733910	Putative potassium channel protein	61.8/5.98	67-74/4.9-5.1	14	23	*Oryza sativa*			1.3

20	gi|30677910	Rab GTPase homolog A3; GTP binding	26.2/5.52	26-29/5.5-5.6	10	43	*Arabidopsis thaliana*	1.4	1.2	
**Hormone-Related**										
21	gi|75309814	S-adenosylmethionine synthetase 2	43.1/5.55	54-60/5.7-5.8	10	27	*Pinus contorta*	1.2	2.4	
22	gi|37051113	S-adenosylmethionine decarboxylase	30.6/5.07	32-39/6.0-6.2	6	18	*Pisum sativum*	1.3	2.4	2.3
23	gi|10185572	Zeta-carotene desaturase precursor	47.4/8.82	43-48/4.2-4.6	8	22	*Oryza sativa*	-2.2		-2.1
24	gi|974782	Cobalamine-independent methionine synthase	86.7/6.17	85-92/6.1-6.2	11	15	*Solenostemon scutellarioides*	1.3		
25	gi|27903511	GCPE protein	82.0/5.92	78-83/5.6-5.7	15	21	*Catharanthus roseus*	3.0	2.1	
26	gi|9759324	GcpE protein	79.8/5.89	78-83/5.6-5.7	13	17	*Arabidopsis thaliana*	3.0	2.0	
27	gi|9759324	GcpE protein	79.8/5.89	78-83/5.6-5.7	14	18	*Arabidopsis thaliana*	2.3	1.8	
**Defence-/Stress-Related Proteins**										
28	gi|197717669	RAD23	41.4/4.43	48-55/3.8-4.2	5	6	*Pinus sylvestris*	-1.6	-2.0	-2.5
29	gi|15240922	DNA repair protein RAD23	40.0/4.58	51-58/4.3-4.6	3	7	*Arabidopsis thaliana*	-1.8	-2.6	-3.6
30	gi|219819653	Phi class glutathione transferase	24.3/6.34	23-26/5.0-5.9	3	15	*Cathaya argyrophylla*			1.5
31	gi|87132998	Chalcone synthase	43.1/5.75	42-46/5.3-5.5	8	17	*Abies alba*	-1.9		-2.3
32	gi|31043672	Glutamate-cysteine ligase	57.8/6.25	40-44/4.9-5.1	16	29	*Brassica juncea*	-2.3	-1.4	-1.5
33	gi|1350510	Late embryogenesis abundant protein	19.1/5.76	20-23/5.2-5.4	5	15	*Picea glauca*	2.8	1.2	
34	gi|4234953	NBS-LRR-like protein cD7	93.3/5.94	90-95/5.7-5.8	7	5	*Phaseolus vulgaris*		2.2	1.9
35	gi|34099726	Putative NBS-LRR protein C601	25.0/6.47	21-25/5.9-6.1	7	22	*Pinus monticola*	1.2	1.4	1.3
36	gi|34099708	Putative NBS-LRR protein G6261	22.7/5.97	20-25/5.7-5.8	6	16	*Pinus monticola*			
37	gi|46518272	CC-NB-LRR protein	117.4/6	97-105/4.9-5.1	18	21	*Solanum tuberosum*		1.3	
38	gi|18401792	MOS4 (MODIFIER OF SNC1,4)	29.7/5.32	28-31/4.9-5.1	11	33	*Arabidopsis thaliana*	-1.9	-2.4	-1.9
39	gi|18377609	Putative UV-damaged DNA binding factor	30.7/5.58	27-31/4.9-5.2	11	33	*Arabidopsis thaliana*	-1.7	-2.3	-1.9
40	gi|168314	Pollen allergen	27.3/5.38	24-28/4.9-5.2	13	37	*Lolium perenne*		2.1	1.9
41	gi|15240211	CYP93D1; oxygen binding	57.4/6.04	75-81/6.3-6.6	14	27	*Arabidopsis thaliana*	1.9		
42	gi|39939493	Ascorbate peroxidase	27.2/5.40	26-31/5.6-5.7	6	30	*Pinus pinaster*	1.4	1.3	1.2
43	gi|16588758	Abscisic stress ripening-like protein	20.7/5.68	20-24/5.6-5.7	5	17	*Prunus persica*	-1.4	-1.3	1.5
44	gi|30693971	Universal stress protein (USP) family protein	17.8/5.66	18-21/5.2-5.4	4	21	*Arabidopsis thaliana*	4.0	3.3	3.7
45	gi|6466176	Intracellular pathogenesis-related protein	17.8/5.66	18-21/5.2-5.4	9	47	*Picea glauca*	3.5		
46	gi|1477584	Abietadiene cyclase	99.5/5.53	97-99/5.2-5.4	13	17	*Abies grandis*		1.2	
47	gi|15080737	Abietadiene synthase	98.0/5.54	90-97/5.6-5.7	10	13	*Abies grandis*	1.5		
48	gi|15080732	Delta-selinene synthase	67.7/5.37	59-66/4.9-5.1	11	22	*Abies grandis*	-1.7	-3.5	-2.1

49	gi|15080732	Delta-selinene synthase	67.7/5.37	59-66/4.9-5.1	12	20	*Abies grandis*	-1.8	-3.5	-2.8
50	gi|7381253	(-)-limonene/(-)-alpha-pinene synthase	73.2/6.31	72-77/5.9-6.1	11	18	*Abies grandis*	2.6	1.5	
51	gi|1419036	Delta-1-pyrroline-5-carboxylate synthase	81.8/6.26	87.5/5.4-5.6	15	20	*Medicago sativa*	1.7	1.6	
52	gi|123593	Heat shock 70 kDa protein	70.6/5.22	75-83/5.3-5.4	17	28	*Zea mays*			
57	gi|157091244	GRP94	95.3/4.82	94-97/4.6-4.9	16	23	*Pinus taeda*	-1.5	-1.5	-1.4
**RNA Transcription and Processing**										
53	gi|30689925	Transcription elongation factor	80.6/5.15	82-93/4.6-4.9	13	18	*Arabidopsis thaliana*			-2.1
54	gi|7638022	Reverse transcriptase	61.9/6.03	40-43/5.5-5.6	9	16	*Picea glauca*	-2.4	-1.5	-2.1
55	gi|29469813	RNA polymerase beta subunit	79.7/9.04	78-82/6.3-6.6	11	14	*Pinus koraiensis*			-1.7
56	gi|18414951	RNA recognition motif (RRM)-containing protein	33.5/5.85	39-43/4.2-4.6	12	26	*Arabidopsis thaliana*	-2.1	-1.3	-1.7
**Other Metabolism**										
58	gi|1568664	O-methyltransferase	41.8/5.49	53-60/4.6-4.9	8	24	*Pinus radiata*	-1.2	1.6	
59	gi|30580387	Caffeoyl-CoA O-methyltransferase	29.1/5.43	30-38/5.2-5.4	8	28	*Pinus taeda*			2.3
60	gi|930002	Nitrate reductase NR2 (396 AA)	43.6/5.41	50-56/3.8-4.2	10	16	*Arabidopsis thaliana*			
61	gi|9758119	Trigger factor-like protein	65.1/5.22	75-82/4.6-4.9	13	24	*Arabidopsis thaliana*			
62	gi|1183892	Legumin	56.9/6.50	43-47/6.1-6.3	8	18	*Calocedrus decurrens*	2.1	1.8	
63	gi|32186924	Starch branching enzyme IIb	20.2/5.69	25-28/5.5-5.6	7	55	*Sorghum bicolor*	1.5	-1.8	1.3
64	gi|30696227	MSL3 (MSCS-LIKE 3)	74.1/8.58	71-76/6.6-7.0	13	15	*Arabidopsis thaliana*	2.7	2.1	2.2
65	gi|31712106	Putative gag/pol polyprotein	72.5/8.70	62-67/6.3-6.7	14	21	*Oryza sativa*	2.2		
66	gi|42572991	Oxysterol-binding family protein	58.1/5.52	68-74/4.9-5.1	11	25	*Arabidopsis thaliana*	-1.3	-1.4	-1.9
67	gi|8134570	5-methyltetrahydropteroyltriglutamate-homocysteine methyltransferase	84.8/6.1	90-97/6.1-6.3	10	16	*Catharanthus roseus*	1.4		
68	gi|15230992	Meprin and TRAF homology domain-containing protein	37.8/5.67	34-38/5.4-5.6	7	26	*Arabidopsis thaliana*	-2.1	-2.9	-4.4
**Unclassified and Unknown**										
69	gi|115437604	Os01g0558600	22.5/5.15	22-27/4.9-5.1	9	46	*Oryza sativa*	-1.8	-2.3	-1.8
70	gi|52076095	Unknown protein	57.8/8.84	55-61/6.2-6.4	13	25	*Oryza sativa*		1.9	2.2
71	gi|224284393	Unknown	71.3/5.07	53-60/3.9-4.3	17	33	*Picea sitchensis*			
72	gi|9294229	Unnamed protein product	82.4/6.32	82-88/5.8-6.0	15	21	*Arabidopsis thaliana*	2.0	1.6	
73	gi|116791055	Unknown	26.9/4.53	25-28/4.2-4.7	8	27	*Picea sitchensis*		-1.9	1.8
74	gi|116782877	Unknown	22.8/9.16	20-25/6.6-6.9	8	39	*Picea sitchensis*	-1.8	-1.8	
75	gi|15231750	Unknown protein	24.9/6.86	26-29/6.2-6.3	8	30	*Arabidopsis thaliana*		-2.6	-2.5

Abbreviations: MM, Molecular Mass; pI, Isoelectric Point; NP, the Number of Matched Peptides; C, Sequence Coverage for the Identified Protein; DAC, Days After Cutting. The Fold Change is calculated compared 25-5 with 23-12, “-” Before the number indicates down-regulation
