# Supplementary material for: Transcriptome and proteome profiling of adventitious root development in hybrid larch (Larix kaempferi × Larix olgensis)
Source: BMC Plant Biol. 2014 Nov 26;14:305. doi: 10.1186/s12870-014-0305-4 (PMC4253636; doi:10.1186/s12870-014-0305-4)
Supplement: Additional file 4: — Concentration and quality of total RNA extracted from materials used in this study. [file 12870_2014_305_MOESM4_ESM.pdf]

Table 1 The concentration and quality of total RNA which were determined by a NanoDrop 1000.

Samples	Before DNase treated		Yield	After DNase treated		Yield
	Absorbance ratio		ug/g	Absorbance ratio		μg/g
	A260/A230	A260/A280		A260/A230	A260/A280	
A	1.67	2.02	698.23	2.17	2.06	397.63
B	1.76	1.98	820.16	2.20	2.03	437.97
C	1.72	2.03	825.38	2.15	2.06	415.58
D	1.68	2.00	670.55	2.19	2.06	330.59

A: 23-12 at 14 DAC (days after cutting), B: 25-5 at 14 DAC, C: 23-12 at 25 DAC, D: 25-5 at 25 DAC.

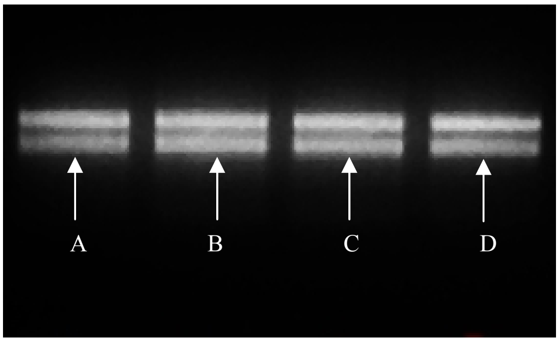

Figure 1 The electrophoregram of four samples on 1% agarose gels.
