# Supplementary material for: Transcriptome and proteome profiling of adventitious root development in hybrid larch (Larix kaempferi × Larix olgensis)
Source: BMC Plant Biol. 2014 Nov 26;14:305. doi: 10.1186/s12870-014-0305-4 (PMC4253636; doi:10.1186/s12870-014-0305-4)
Supplement: Additional file 5: — The detailed information of normalization. [file 12870_2014_305_MOESM5_ESM.pdf]

Normalization between the libraries

1 GO classification

The total unigenes detected in GO annotation in each library are showed in the following table.

The number of unigenes in GO annotation in each library

	23-12/14DAC	25-5/14DAC	23-12/25DAC	25-5/25DAC
Total	18652	13708	16690	16967

When we compare 14DAC to 25DAC of same clone in GO categories, in order to avoid the effect of systematic bias, we do normalization between 23-12/14DAC and 23-12/25DAC, 25-5/14DAC and 25-5/25DAC separately. The number of unigene in all GO categories in 23-12/14DAC library divide by 1.1 (18652/16690=1.1), in 25-5/25DAC library divide by 1.2 (16967/13708=1.2). The normalized data has been drawn the histogram of GO classifications (figure 3 and 4).

2 The number of reads in each library

The total read number got from the each library is showed in the following table.

The total read number of each library

	23-12 14DAC	25-5 14DAC	23-12 25DAC	25-5 25DAC
Total read number	239163	182468	279438	256763

When we compare 25-5 to 23-12 at same development stage in table 1, in order to avoid the effect of systematic bias, we do normalization between 25-5/14DAC and 23-12/14DAC, 25-5/25DAC and 23-12/25DAC separately. The read number in 23-12/14DAC library divide by 1.3 (239163/182468=1.3), in 23-12/25DAC library divide by 1.09(279438/256763=1.09). The normalized data has been showed in table 1.
